# Radiation-Induced Cancer Risks from Computed Tomographic Scans During COVID-19 Pandemic

**DOI:** 10.5152/eurasianjmed.2022.21114

**Published:** 2022-06-01

**Authors:** Hadi Majidi, Seyed Jalal Hosseinimehr

**Affiliations:** 1 Department of Radiology, Mazandaran University of Medical Sciences Faculty of Medicine, Sari, Iran; 2 Department of Radiopharmacy, Mazandaran University of Medical Sciences Faculty of Pharmacy, Sari, Iran

The coronavirus disease 2019 (COVID-19) is a highly severe respiratory disease that has been spreading globally and has been stated as a pandemic by World Health Organization (WHO). Chest computed tomography (CT) scan has been suggested to diagnose COVID-19 disease with significant sensitivity, and even it is utilized for screening asymptomatic patients. Computed tomography scan has been widely used for diagnosis of COVID-19,^1^ due to its availability in most of the central hospitals in the city. Computed tomography is an important complement to polymerase chain reaction (PCR) for the diagnosis of COVID-19. Computed tomography scan has high sensitivity (67-100%) and relatively low specificity (25-80%).^2,3^ The rapid increase in CT utilization was observed at the early diagnosis and management of patients due to increased outbreak of COVID-19 in the world. First, we studied the number of chest CT scans 7 months before the COVID-19 pandemic (from February 20, 2019, to September 22, 2019) and compared it to the same duration during the COVID-19 pandemic (from February 20, 2020, to September 22, 2020) in our hospital (Imam Hospital, Sari, Iran). The number of lung CT scans was 3044 before the COVID-19 pandemic, while it was 14 048 during the COVID-19 pandemic for the same duration. A significantly increased number of CT scans (460%) have been performed in the radiology ward of this hospital. It is well documented that a similar increased number of chest CT scans has been performed in other clinics or hospitals in the world. While a 2-fold increase in the number of CT scans from 1997 to 2008 was observed in the United Kingdom,^4^ there was a 50% increase from 2000 to 2006 in United States.^5^

The public has been exposed to radiation mainly from natural background and then from medical imaging. An increased mean annual dose per person from medical imaging has been observed in the population. The risk of radiation-induced cancer was estimated from radiation exposure diagnostic instruments. Computed tomography scans expose higher radiation doses to patients as compared to conventional diagnostic x-rays such as chest x-ray. The estimated effective doses are in the range of 1-10 mSv from diagnostic CT procedures. The mean effective dose from chest CT procedure is typically estimated as 10 mSv, while some of the Japanese survivors of the atomic bombs received the estimated lowest doses of 5-20 mSv. These survivors are estimated to receive these doses slightly larger than CT scans. They have demonstrated a small but increased radiation-related excess cancer risk. However, the low-dose protocol CT scan was performed in some clinical COVID-19 diagnosis with a dose of about 0.2 mSv that reduced the patient’s dose to 1/8 to 1/9 of the standard dose.^6^ Sometimes, 3-6 CT scans within a short period of time are performed for a patient with COVID-19 disease.^7^ The large-scale utilization of chest CT in identifying COVID-19 would significantly enhance the radiation exposure of the public.^3^

The biological effects of ionizing radiation VII lifetime risk model predict that a CT procedure with an effective dose of 10 mSv may be associated with an increase in cancer risk of approximately 1 in 1000 CT examinations and fatal cancer of approximately 1 in 2000.^5,8^ A nonrandomized, low-dose CT scan, lung cancer screening trial was performed in Italy from 2004 to 2015. It showed that the numbers of lung and major cancer cases induced by CT scans during 10 years of screening were 1.5 and 2.4 in this cohort, respectively. It showed an additional risk of radiation-induced cancers of 0.05% (2.4/5203). In this study, 259 lung cancers were diagnosed by screening; one case of radiation-induced cancer would be expected for every 108 lung cancers that were detected through screening by CT scans.^9^ The additional risk of cancer after CT scan exposure in the screening study was mostly due to radiation exposure. The absolute additional all-cancer risks were 9.38 per 100 000 person-years. The average effective radiation dose per scan was estimated as 4.5 mSv.^10^ The repeated CT screening increases cancer risk from irradiation. The smallest cancer risks are reported for repeated mammographies and were estimated as 0.3-0.6 cases per 1000 women who are screened every 3 years due to the low-radiation dose exposure per x-ray during mammography. Breast is the particularly received measurable dose to organ in mammography. The highest incidence rate for cancer risk was reported for lung CT screening and was estimated as 2.9-8.0 per 1000 in females. A total of 5-7 radiation-induced cancers per 1000 men and 6-13 cancers per 1000 women were estimated if individuals performed all of these screening examinations routinely over their lifetime. Radiation-induced cancer risks are higher for women than for men due to breast exposure to radiation.^4^ According to risk models in the National Research Council Biological Effect of Ionizing Radiation report, about 4100 cases of lung cancer annually could be associated with CT examinations in the US population.^11^

While CT examinations can provide great medical benefit for the diagnosis of the respiratory symptoms in patients with COVID-19, great attention is needed to potential future radiation-induced cancer risks because of the dramatic increase in the number of chest CT scans in public. In previous cohort studies, a doubling in the number of CT examinations was reported in 10 years, while a 4.6-fold increase in the number of chest CT scans has been reported during the COVID-19 pandemic in 7 months duration. The radiation risks have to be considered for judging the feasibility of chest CT in COVID-19 screening. The risk–benefit ratio should be considered when chest CT is used as a first-line screening tool in a large population. The cancer risks to patients under chest CT scans are likely to be small, but because of the large number of persons exposed to radiation due to COVID-19 disease, in addition, repeated lung CT examinations are significantly more than routine annually CT scans, even small risks could come along a considerable number of future cancers. For example, if there is one new case of cancer per 1000 chest CT scans in a public and now if the number of needed chest CT scans have been increased more than 4 times in that public, then it is suggested that the probability of new cancer case will be increased significantly in future. It is necessary to perform the risk–benefit analyses on the number of radiation-induced cancers and number of potentially diagnosed COVID-19 diseases and lung involvements that were discovered by CT scans. With the importance of the wide use of lung CT scans in patients with COVID-19 disease, it is necessary to estimate radiation-related cancer risk in the population during the COVID-19 pandemic of future cancer risks from current CT scan use. Although clinical benefits for COVID-19 diagnosis should overcome the small radiation-induced cancer risks from CT scans, they should be kept as low as possible with a modified low-dose imaging protocol. In addition, alternative COVID-19 diagnosis procedures should be considered to use if appropriate, which do not involve exposure to ionizing radiation. In addition, repeated CT scans should be avoided in patients in a short time if possible. We should be cautious in the use of chest CT screening in asymptomatic cases who are outside the COVID-19 epidemic center and in young women and children. By the way, CT instrument is expensive, and the extremely high utilization of CT instrument results in the depreciation of this machine and thus, a heavy financial burden for hospital management and the National Health System particularly in low- and middle-income countries.
